# ARDS and aging: TYMS emerges as a promising biomarker and therapeutic target

**DOI:** 10.3389/fimmu.2024.1365206

**Published:** 2024-03-15

**Authors:** Gang Li, Ke Yan, Wanyi Zhang, Haiyan Pan, Pengxiang Guo

**Affiliations:** ^1^ Department of Emergency Medicine, The Third Affiliated Hospital of Zhejiang Chinese Medical University, Hangzhou, Zhejiang, China; ^2^ School of Life Sciences, Beijing University of Chinese Medicine, Beijing, China; ^3^ Department of Pharmacology of Chinese Materia Medica, School of Traditional Chinese Pharmacy, China Pharmaceutical University, Nanjing, China

**Keywords:** ARDS, aging, TYMS, WGCNA, machine learning, immune infiltration, snRNA-seq

## Abstract

**Background:**

Acute Respiratory Distress Syndrome (ARDS) is a common condition in the intensive care unit (ICU) with a high mortality rate, yet the diagnosis rate remains low. Recent studies have increasingly highlighted the role of aging in the occurrence and progression of ARDS. This study is committed to investigating the pathogenic mechanisms of cellular and genetic changes in elderly ARDS patients, providing theoretical support for the precise treatment of ARDS.

**Methods:**

Gene expression profiles for control and ARDS samples were obtained from the Gene Expression Omnibus (GEO) database, while aging-related genes (ARGs) were sourced from the Human Aging Genomic Resources (HAGR) database. Differentially expressed genes (DEGs) were subjected to functional enrichment analysis to understand their roles in ARDS and aging. The Weighted Gene Co-expression Network Analysis (WGCNA) and machine learning pinpointed key modules and marker genes, with ROC curves illustrating their significance. The expression of four ARDS-ARDEGs was validated in lung samples from aged mice with ARDS using qRT-PCR. Gene set enrichment analysis (GSEA) investigated the signaling pathways and immune cell infiltration associated with TYMS expression. Single-nucleus RNA sequencing (snRNA-Seq) explored gene-level differences among cells to investigate intercellular communication during ARDS onset and progression.

**Results:**

ARDEGs are involved in cellular responses to DNA damage stimuli, inflammatory reactions, and cellular senescence pathways. The MEmagenta module exhibited a significant correlation with elderly ARDS patients. The LASSO, RRF, and XGBoost algorithms were employed to screen for signature genes, including CKAP2, P2RY14, RBP2, and TYMS. Further validation emphasized the potential role of TYMS in the onset and progression of ARDS. Immune cell infiltration indicated differential proportion and correlations with TYMS expression. SnRNA-Seq and cell-cell communication analysis revealed that TYMS is highly expressed in endothelial cells, and the SEMA3 signaling pathway primarily mediates cell communication between endothelial cells and other cells.

**Conclusion:**

Endothelial cell damage associated with aging could contribute to ARDS progression by triggering inflammation. TYMS emerges as a promising diagnostic biomarker and potential therapeutic target for ARDS.

## 1 Introduction

Acute Respiratory Distress Syndrome (ARDS) represents a life-threatening manifestation of severe respiratory failure that can occur in any condition or disease that causes lung injury (1). The pathological changes in ARDS are characterized by diffuse alveolar damage, including damage to alveolar epithelial and pulmonary capillary endothelial cells, inflammation and infiltration of immune cells, widening of interstitial edema, protein-rich edema fluid in the alveoli, fibrous protein deposition, formation of transparent membranes, and microthrombosis (2). These changes lead to dysregulation of the ventilation/perfusion (V/Q) ratio, impaired gas exchange, a significant increase in intrapulmonary shunting, decreased lung compliance, reduced lung volume (baby lung), and ultimately acute hypoxemic respiratory failure (3). The mortality rate of ARDS is as high as 40% (4), making it one of the main reasons for patients to be transferred to the ICU (5). Based on statistics, the incidence rate of ARDS is several tens of cases per 100,000 person-years (6). Numerous studies have shown that elderly patients account for a high proportion of ARDS patients, and age is a significant risk factor affecting the development and prognosis of ARDS (7). Currently, the diagnosis of ARDS mainly relies on clinical manifestations, but the problem of missed diagnosis and delayed diagnosis remains unresolved. Therefore, studying the molecular biological mechanisms of ARDS and identifying potential biomarkers is of great significance for early recognition, diagnosis, and assessment of the severity and prognosis of ARDS.

Increasing evidence suggests that the pathogenesis of ARDS involves multiple biological functions, including inflammatory response (8), oxidative stress (9), apoptosis (10, 11), and endoplasmic reticulum autophagy (12). Amidst these factors, aging is pivotal in the genesis and advancement of ARDS. Aging is a complex and multidimensional process that leads to widespread organ dysfunction (13) and various age-related diseases (14), such as neurodegenerative disorders, diabetes, idiopathic pulmonary fibrosis, etc. (15). DNA damage serves as a primary catalyst for aging, whereas DNA repair acts as a pivotal determinant of aging. Additionally, deficiencies in DNA repair can accelerate the progression of various age-related diseases (16). Lung aging is associated with molecular and physiological changes that result in altered lung function, impaired lung remodeling and regeneration, and increased susceptibility to acute and chronic lung diseases (17). Older patients with sepsis have worse outcomes, which may be related to the decline in immune system function and changes in the pulmonary vascular system in the elderly (18, 19). Immune cell activation is a major mediator of ARDS inflammation, and immunosenescence may affect the pathogenesis and prognosis of elderly ARDS subgroups (20). A cross-age study of ARDS patients showed a correlation between age and neutrophil biomarker myeloperoxidase (MPO) in bronchoalveolar lavage fluid (BALF) (21). Compared with younger groups, Tregs and inflammatory markers increased in older groups (22). Aging is implicated in various pathways of ARDS pathogenesis, yet the precise mechanisms remain elusive. Therefore, further research is needed to understand the relationship between cellular aging, immune infiltration landscape, and their interplay in the context of ARDS disease.

Over the past decade, high-throughput sequencing has experienced rapid development and has permeated various fields of life sciences, significantly advancing the progress of basic medical research (23). GSE163426 is a dataset related to ARDS, containing a large number of samples (47 ARDS patients and five control individuals). In this study, we used various bioinformatics methods to obtain age-related characteristic genes for ARDS. Four hub ARDS-ARDEG genes demonstrated significant diagnostic performance and were validated in external datasets. Additionally, we corroborated the expression levels of the selected genes through animal experiments. Thymidylate Synthetase (TYMS) was identified as a promising diagnostic biomarker for ARDS in aging individuals. We assessed the enriched signaling pathways associated with TYMS expression and involvement in immune cell infiltration. Single-nucleus RNA sequencing highlighted elevated TYMS expression in endothelial cells, indicating its potential role in endothelial dysfunction seen in ARDS. These findings highlight TYMS’s multifaceted involvement, particularly its modulation of immune responses and contribution to endothelial proliferation and vascular injury repair, presenting TYMS as a potential therapeutic target for precise intervention in ARDS among the elderly ICU population.

## 2 Materials and methods

### 2.1 Data sources

In our study, we acquired two bulk RNA sequencing (bulk-RNAseq) datasets, specifically GSE163426 and GSE84439, from the GEO database. The GSE163426 dataset was utilized as the training set, encompassing 47 ARDS patients and five control individuals. The validation group, comprising seven sepsis patients and 8 ARDS patients, was derived from the GSE84439 dataset. Additionally, we utilized publicly available single-nucleus RNA sequencing (snRNA-Seq) data obtained from autopsy lung tissues of 19 COVID-19 patients and seven healthy donors. These data, including the corresponding clinical information, can be accessed at the GEO database using the accession number GSE171524.

### 2.2 Downloading and organizing aging-related genes

In order to identify ARGs for our study, we downloaded data from the HAGR database (https://genomics.senescence.info/) (24). This database comprises the GenAge dataset (307 genes) and the CellAge dataset (949 genes). After combining these datasets and eliminating duplicate genes, we obtained 1256 ARGs for subsequent analysis.

### 2.3 Identification of aging-related DEGs

To identify ARDEGs, we utilized the R package limma for differential analysis (25). The filtering criteria were set as |logFC|>1 and *P*<0.05. This analysis was performed on the ARDS and control samples. The intersection of the DEGs with the ARGs resulted in the identification of ARDEGs.

### 2.4 Construction of PPI network

Using the Search Tool for the Retrieval of Interacting Genes (STRING) database, we constructed a protein-protein interaction (PPI) network for evaluating the gene interactions among ARDEGs (26).

### 2.5 Functional and pathway enrichment analysis

The Kyoto Encyclopedia of Genes and Genomes (KEGG) and Gene Ontology (GO) were used in the functional enrichment analysis of ARDEGs (27). The GO analysis involved exploring the biological processes (BP), cellular components (CC), and molecular functions (MF) associated with these ARDEGs.

### 2.6 Weighted gene co-expression network analysis

The WGCNA analysis was conducted to construct a co-expression network in the GSE163426 dataset, utilizing the scale-free topology criterion (28). The dynamic tree-cutting method with a minimum module size of 30 was used to identify co-expressed gene modules. Gene significance (GS) values and module membership (MM) values were employed to assess the association between gene modules and both ARDS and aging, ultimately determining the key modules.

### 2.7 Identification of hub ARDS-ARDEGs

The least absolute shrinkage and selection operator (LASSO), Random Forest (RF), and Extreme Gradient Boosting (XGBoost) algorithms were employed as the methods for screening and identifying key ARDS-ARDEGs. LASSO analysis was performed with the glmnet program, and 10-fold cross-validation was used to assess the penalty parameter (29). This approach outperforms traditional regression analysis methods in the assessment of high-dimensional data. XGBoost, a machine learning algorithm based on gradient boosting trees, was employed to assess the importance of ARDEGs in both ARDS and aging (30). The R package XGBoost aided in determining the significance of models constructed with various sets of ARDEGs. The random forest algorithm with recursive feature elimination (RFE) is a supervised machine learning method (31). The RFE approach was conducted by setting the number of decision trees to 500 and identifying aging-associated signature genes with relative importance greater than one. The intersection of aging-associated signature genes obtained through three machine learning filters using the R package Venn was defined as hub ARDS-ARDEGs. To assess the diagnostic utility of hub ARDS-ARDEGs in aging ARDS patients, the receiver operating characteristic curve (ROC) analysis was performed on the GSE1919 and GSE89408 datasets.

### 2.8 Experimental animals

This study was conducted in accordance with ethical guidelines and received ethical approval from the Ethics Committee of Hangzhou Hibio Technology Co., Ltd. (HB2311003). C57BL/6 mice, including males and females, were procured from Jiangsu Jiangsu Huachuang sino Pharma Tech Co., Ltd. The study utilized young mice (3 months old) and aged mice (18 months old). The ARDS mouse model was induced by intraperitoneal injection of lipopolysaccharide (LPS) (Sigma-Aldrich) at 5mg/kg for young mice and 2.5mg/kg for aged mice. Following 72 hours of LPS administration, the mice were euthanized, and lung tissues were collected for subsequent analysis.

### 2.9 Histopathological observation of lung tissue

Lung tissue was collected from the middle lobe of the right lung in mice. Following alcohol dehydration, paraffin embedding, and sectioning, the samples were stained with H&E and subsequently examined under an optical microscope to evaluate the extent of lung tissue damage. The freshly removed lung tissue from the left lobe was weighed using an analytical scale to obtain the wet weight (W). The tissue was then dried in an oven until a consistent weight was reached to calculate the dry weight (D). To determine the degree of pulmonary edema, the formula W/D × 100% was applied to obtain the wet-to-dry weight ratio.

### 2.10 Quantitative real-time PCR

Reverse transcription of total RNA into complementary DNA (cDNA) was performed using the TOROBlue^®^ qPCR RT Kit after RNA extraction from the upper lobe of the right lung was performed using Trizol (Vazyme). TOROGreen^®^qPCR Master Mix was used for qRT-PCR. The primer sequences for hub ARDS-ARDEGs are presented in **Table S9** of the **Supplementary Materials**. Internal reference gene GAPDH was employed. Each group included six biological samples.

### 2.11 Analysis of SnRNA-seq data

The raw matrix in each dataset was normalized using the “LogNormalize” function in Seurat (version 4.3.1) (32). The “RunHarmony” function in the “harmony” package was utilized to reduce the batch effect (33). The “FindVariableFeatures” function was employed to select the top 2000 most variable genes as input data. Principal component analysis was used to determine the 19 best principal components for data integration. Following the acquisition of principal components, several cell types were distinguished using established cell-specific markers, and uniform manifold approximation and projection were utilized to view the cells.

### 2.12 Cell–cell communication analysis

Based on ligand-receptor interactions, cell-cell communication between several cell clusters was inferred and visualized using the R software’s “CellChat” package (34). The “CellChat” tool was used to import the snRNA-seq data that had been normalized using the “Seurat” package. We focused on the secreted signaling pathways and analyzed the communication between all cell types. Moreover, endothelial cells acted as signal senders and receivers in our particular analysis and visualization of the communication between these cells and other cell types.

### 2.13 Statistical analysis

R software (version 4.3.1) was used to conduct statistical analysis. Two groups were compared using Wilcoxon tests. The association between immune cell infiltration and hub ARDS-ARDEGs expression levels was examined using Spearman correlation analysis. A statistical significance threshold of *P* < 0.05 was applied. The mean ± standard deviation of a minimum of six independent experiments was used to present the statistical analysis of the qRT-PCR data, and unpaired two-tailed Student’s t-tests were employed to evaluate differences (*P < 0.05; **P < 0.01; ***P < 0.001). For statistical significance, a *P*-value of less than 0.05 was used.

## 3 Results

### 3.1 Identification of ARGEGs

**Figure 1:**
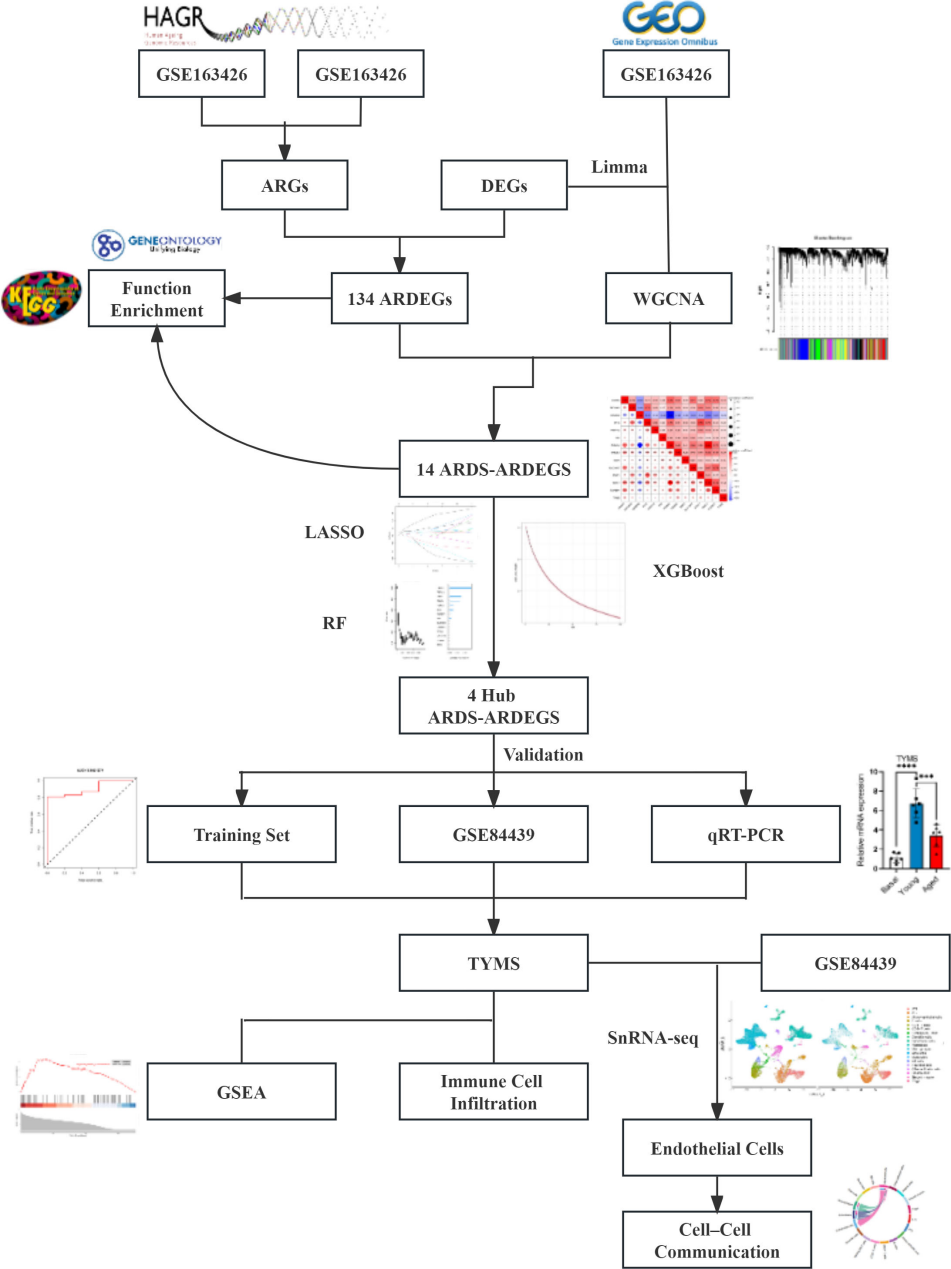
The flowchart outlining the methodology of this investigation.

**Figure 2:**
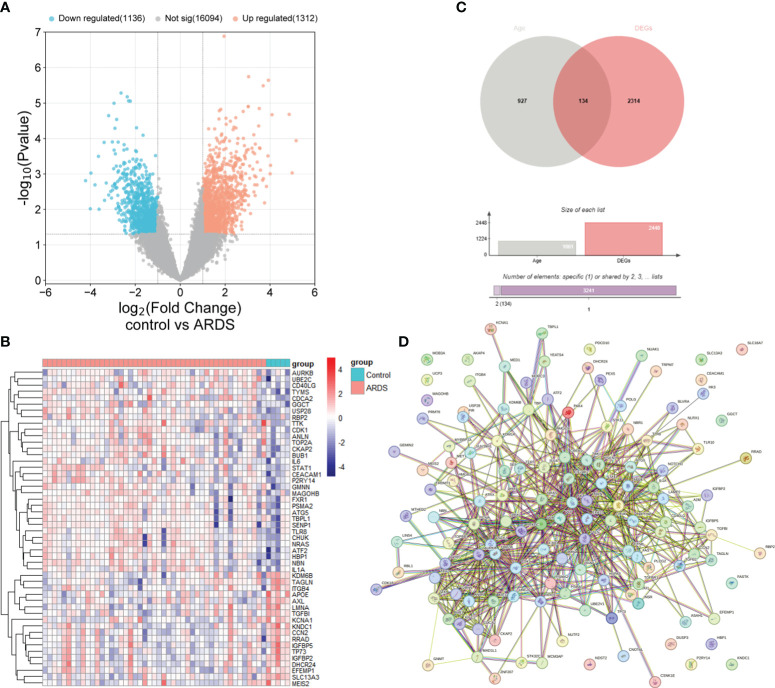
Identification of ARDEGs between control and ARDS. **(A)** The differential gene expression between the control and ARDS groups is displayed using a volcano plot. **(B)** A Venn diagram illustrates the overlap between ARGs and DEGs in the ARDS and control groups. **(C)** A heatmap is presented illustrating the expression patterns of the 50 most significant ARDEGs, based on the smallest *p*-values, in both the control and ARDS groups. **(D)** An interaction network map shows protein interactions among the 134 ARDEGs.

The study’s procedural diagram is depicted in **Figure 1**. A total of 2448 DEGs between control and ARDS tracheal aspirates were identified, with 1312 genes up-regulated and 1136 genes down-regulated (**Figure 2A**). 134 ARDEGs were obtained by the intersection of ARGs and DEGs (**Figure 2B**; **Supplementary Table S1**). Using the R program limma, expression matrices of ARGs were taken out of the training set, and their differences were examined. A gene expression heat map was created to see the top 50 genes with the most significant differences (**Figure 2C**). A close association between ARDEGs at the protein level was found by the PPI protein network analysis (**Figure 2D**; **Supplementary Table S2**).

### 3.2 Functional enrichment analysis of ARDEGs

**Figure 3:**
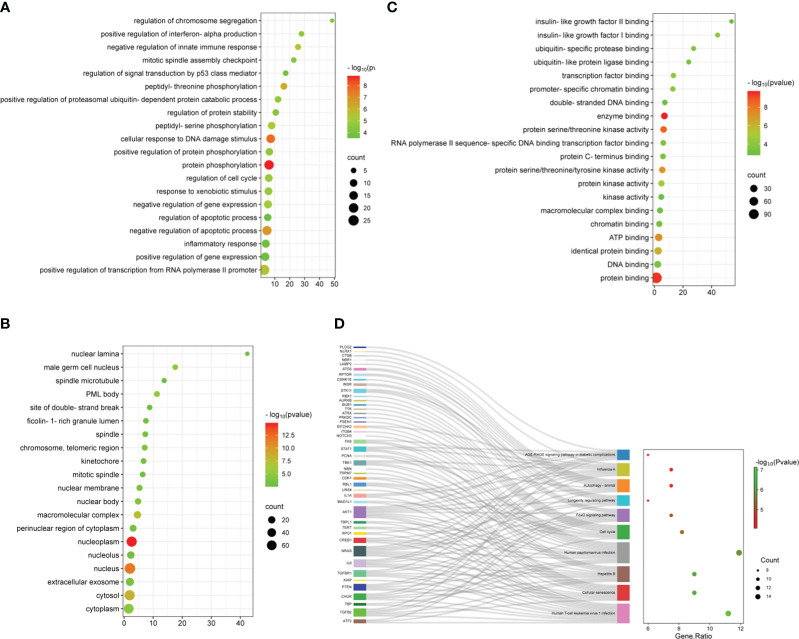
Functional enrichment analysis of ARDEGs. **(A)** Depicted are the outcomes of GO enrichment analysis for BP, **(B)** CC, and **(C)** MF. The bubble plot illustrates the top 20 significantly enriched functions, with bubble size representing the number of DEGs (larger circles indicate more DEGs) and color representing the adjusted *p*-value (redder colors indicate smaller *p*-values). **(D)** KEGG enrichment analysis results are visualized in a Sankey dot pathway enrichment plot, displaying the top 10 significantly enriched pathways.

To understand the potential mechanisms of ARDEGs in ARDS and aging, we employed the R package clusterProfiler to perform GO and KEGG enrichment studies on ARDEGs (**Supplementary Tables S3**, **4**). The GO enrichment analysis in BP showed that the first five ARDEG enrichments were primarily associated with protein phosphorylation, cellular response to DNA damage stimulus, negative regulation of apoptotic process, peptidyl-threonine phosphorylation, and positive regulation of transcription from RNA polymerase II promoter (**Figure 3A**). The top 20 enriched elements in MF and CC are shown in **Figures 3B, C**. Furthermore, these ARDEGs were considerably enriched in Cellular senescence, Cell cycle, FoxO signaling pathway, Longevity regulating pathway, and Autophagy (**Figure 3D**).

### 3.3 Construction of the weighted gene co-expression network

**Figure 4:**
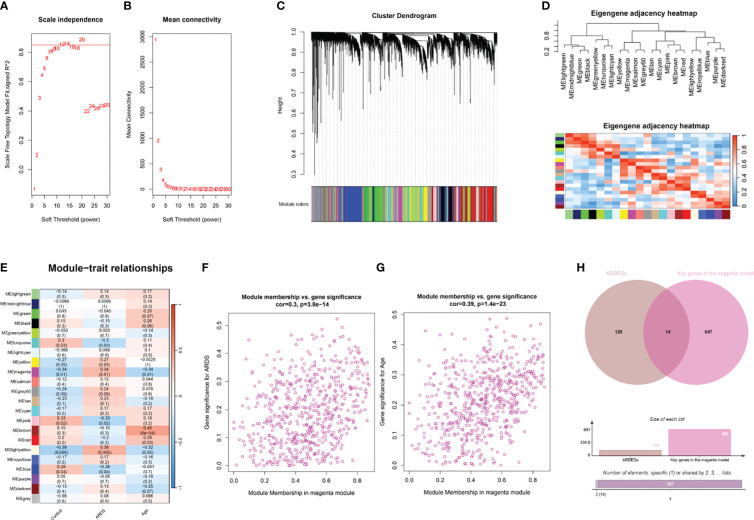
WGCNA analysis and identification of ARDS-ARDEGs in GSE163426. **(A)** The soft thresholding power of WGCNA is shown. **(B)** The average connectivity of WGCNA is displayed. **(C)** The top 75% of the gene clustering tree’s variance is displayed, with each gene represented by a branch and a co-expression module by a color underneath. **(D)** The association of feature genes between modules is displayed on a heatmap. **(E)** A heatmap illustrating the correlations between the modules and traits is displayed; each color corresponds to a co-expression module, and the numbers indicate the *p*-values and module-trait correlation coefficients. **(F)** The relationship between the ARDS GS and the magenta module’s MM is displayed as a scatter plot. **(G)** A scatter plot illustrates the relationship between the magenta module’s MM and aging GS. **(H)** The intersection of ARDEGs along with significant genes in the magenta module is shown in a Venn diagram.

The R package WGCNA was employed for co-expression network construction, utilizing the variance in gene expression of the initial 75% of genes as a screening criterion. A total of 13,906 genes were encompassed in this network assembly. The determination of the soft threshold power at 12 yielded a scale-free index of 0.85, along with notably favorable mean connectivity, enabling the establishment of a scale-free network (**Figures 4A, B**). Following this, cluster analysis was conducted to identify highly analogous modules, resulting in the display of the cluster dendrogram (**Figure 4C**). The correlation among these modules was calculated (**Figure 4D**). The findings revealed a notable correlation between the MEmagenta module and ARDS (cor=0.34; *p*=0.01), as well as aging (co=-0.34; *p*=0.01) (**Figure 4E**; **Supplementary Table S5**). Moreover, a significant correlation between MM and GS for ARDS within the magenta module (cor=0.3; *p*=3.8e-14) was observed, as well as aging (co=0.39; *p*=1.4e-23) (**Figures 4F, G**). The genes in the magenta module were selected as the candidate genes and compared with ARDEGs, resulting in the identification of 14 genes that were both in the magenta module and associated with ARDS and aging (**Figure 4H**).

### 3.4 Correlation and enrichment analysis of ARDS-ARDEGs

**Figure 5:**
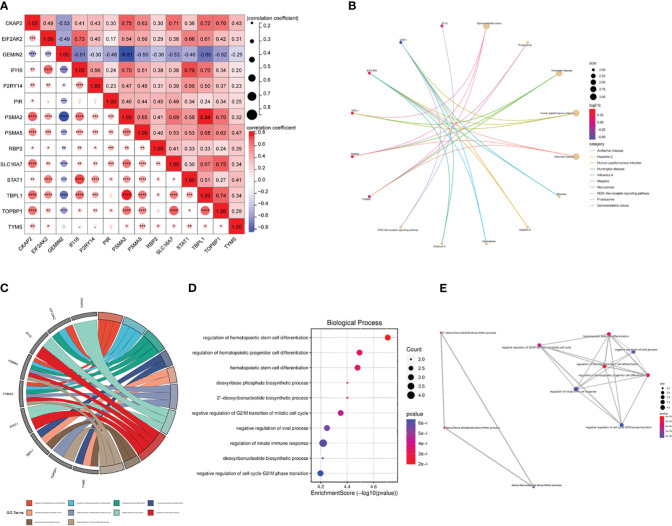
The correlation and enrichment analysis of ARDS-ARDEGs. **(A)** The ARDS-ARDEG correlation analysis is shown, with statistical significance denoted by *P<0.05, **P<0.01, and ***P<0.001. **(B)** A circular plot illustrates the top 10 entries for KEGG enrichment analysis of ARDS-ARDEGs. **(C)** A chord plot shows the top 10 BP entries for GO enrichment. **(D)** A bubble plot is presented, displaying the BP entries for GO enrichment. **(E)** A network plot indicates the BP entries associated with the ARDS-ARDEG analysis.

We used the Pearson correlation coefficient to assess the relationship between the ARDS-ARDEGs. PSMA2 exhibited a high correlation with TTBPL1 (cor=0.84) (**Figure 5A**). The KEGG pathway enrichment analysis demonstrated that the top ten enriched pathways associated with ARDS-ARDEGs were primarily involved in Spinocerebellar ataxia, Proteasome, Measles, Hepatitis C, Necroptosis, Influenza A, and the NOD-like receptor signaling pathway (**Figure 5B**; **Supplementary Table S6**). According to the GO enrichment analysis, ARDS-ARDEGs are enriched in BP, including the regulation of hematopoietic stem cell differentiation, deoxyribose phosphate biosynthetic process, negative regulation of G2/M transition of the mitotic cell cycle, regulation of the innate immune response, and deoxyribonucleotide biosynthetic process (**Figures 5C, D**; **Supplementary Table S7**). The map illustrating the interactions among BP was subsequently constructed (**Figure 5E**).

### 3.5 Identification of hub ARDS-ARDEGs via machine learning

**Figure 6:**
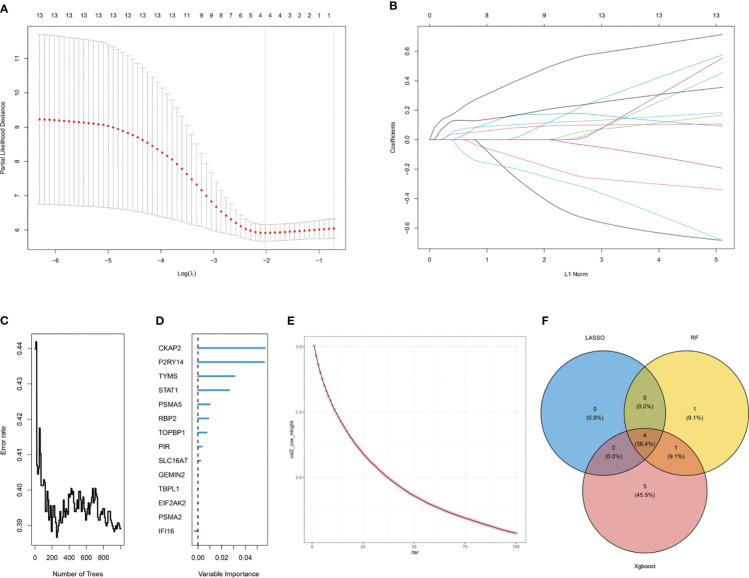
Selection of hub ARDS-ARDEGs using machine learning. **(A)** LASSO coefficient computation. The vertical dashed line shows the ideal lambda value. **(B)** Ten-fold cross-validation for LASSO model parameter adjustment. Every curve represents a gene. **(C)** The correlation between the mistake rate and the number of trees in the random forest. **(D)** Relative relevance ranking for ARDS-ARDEGs. **(E)** XGBoost modeling in the ARDS training set. **(F)** Venn diagram illustrating the intersection of genes selected as aging markers using LASSO, random forest, and XGBoost algorithms.

Three machine learning algorithms were used to screen hub ARDS-ARDEGs in order to identify signature genes in the elderly population with ARDS: LASSO (**Figures 6A, B**), RF (**Figures 6C, D**), and XGBoost (**Figure 6E**). Following the integration of the outcomes from the three algorithms, we ultimately identified four hub genes: CKAP2, P2RY14, RBP2, and TYMS (**Figure 6F**; **Supplementary Table S8**).

### 3.6 Diagnostic efficacy of hub ARDS-ARDEGs

**Figure 7:**
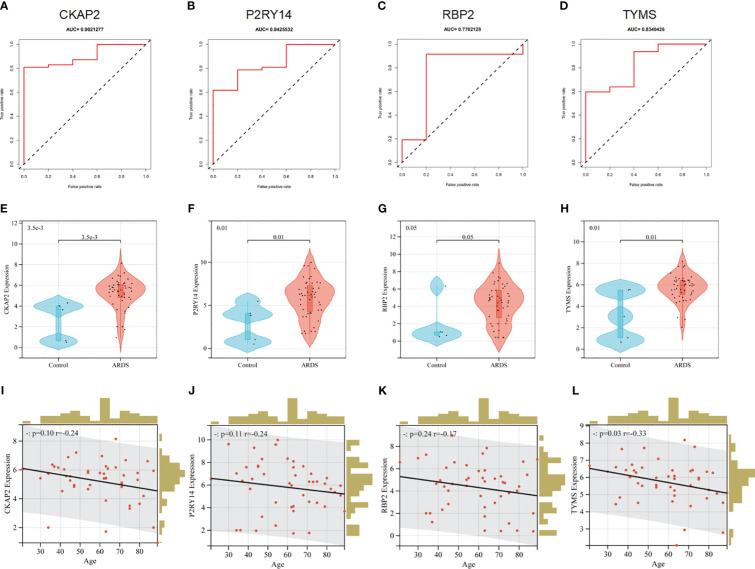
Performance of hub ARDS-ARDEGs in the GSE163426 dataset. **(A-D)** ROC curves demonstrate the chosen genes’ diagnostic efficacy. The AUC is computed to assess the gene’s diagnostic accuracy. **(E–H)** Violin plots show the differences in the expression levels of particular genes between the ARDS patient group and the control group. The control and ARDS patient groups are shown on the x-axis, while the gene expression values are represented on the y-axis. The width of the violin plot represents the density of gene expression values, with a broader plot indicating a higher expression density. **(I-L)** Relationship between the age of ARDS patients and the expression levels of the chosen genes. The x-axis represents the age of ARDS patients, and the y-axis represents the gene expression levels. Each point on the scatter plot represents an individual ARDS patient.

The ROC analysis revealed that the four hubs ARDS-ARDEG had high diagnostic values in the training set. The area under the curve (AUC) of ROC for these signature genes was observed as follows: 0.902 for CKAP2, 0.843 for P2RY14, 0.770 for RBP2, and 0.834 for TYMS (**Figures 7A–D**). Furthermore, the screened signature genes demonstrated higher expression levels in individuals with ARDS than those in the healthy control group, indicating a potential role of these genes in the pathogenesis of ARDS (**Figures 7E–H**). Subsequently, a correlation analysis was conducted between patient age and the expression of hub ARDS-ARDEGs (**Figures 7I–L**). As patient age increased, the expression of these genes decreased, with TYMS demonstrating the strongest correlation with the aging process.

**Figure 8:**
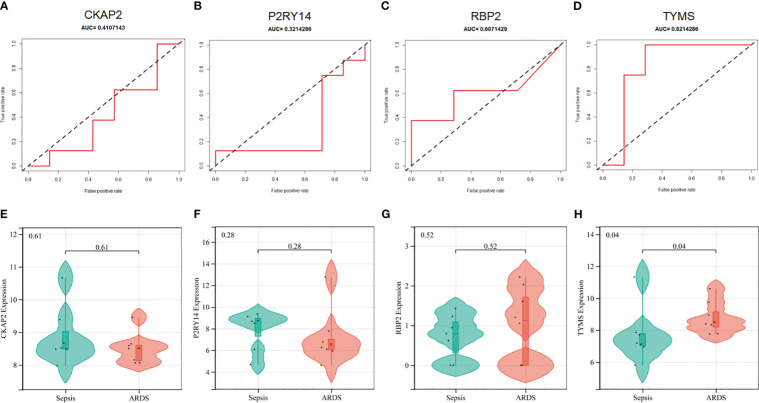
Performance of hub ARDS-ARDEGs in the GSE84439 dataset. **(A-D)** Violin plots compare the expression levels of particular genes in patients with ARDS and those with septic shock. **(E-H)** ROC curves indicate the diagnostic performance of the identified genes about ARDS.

In addition, the diagnostic efficacy of each signature gene in predicting ARDS was evaluated in an external validation cohort. By GSE84439, the AUC values of ROC for CKAP2, P2RY14, RBP2, and TYMS were 0.411, 0.321, 0.607, and 0.821, respectively (**Figures 8A–D**). In comparison to sepsis patients, only TYMS demonstrated significant changes in expression among these characteristic genes in ARDS patients, indicating a remarkable upregulation (**Figures 8E–H**). These observations indicate that TYMS exhibits superior diagnostic efficiency for ARDS, irrespective of whether it is used to distinguish elderly ARDS patients or differentiate sepsis patients.

### 3.7 Aging inhibited TYMS induction in mice after ARDS modeling

**Figure 9:**
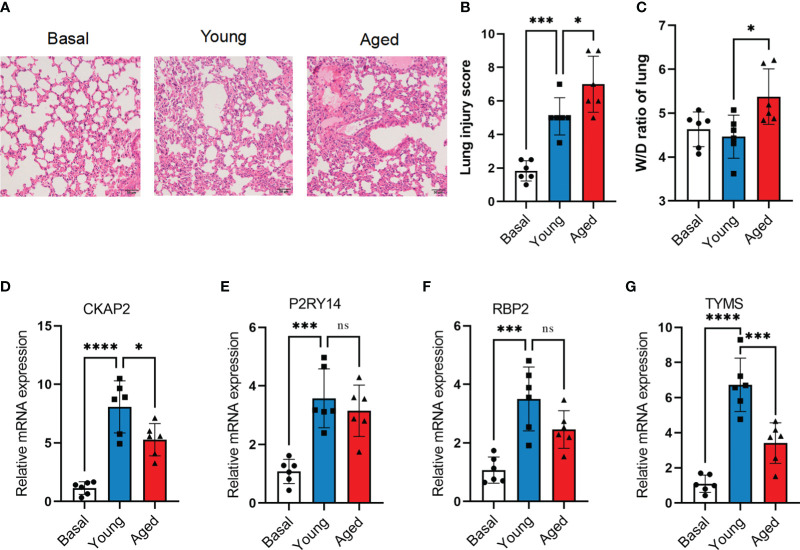
Validation of hub ARDS-ARDEGs through total RNA sequencing. **(A)** Histopathological images of lung tissues in LPS-induced ARDS young and aged mice. Scale bar, 50 μm. **(B)** Lung injury scoring analysis based on alveolar wall congestion and inflammatory cell infiltration, bronchovascular peribronchial hemorrhage and inflammatory cell infiltration, bronchial lumen exudate, and endothelial cell swelling, with assignment of each slide area to a severity score ranging from 1 (average) to 4 (severe injury). **(C)** Lung wet-to-dry ratio in young adult and aged mice 72 hours post LPS exposure. **(D-G)** CKAP2, P2RY14, RBP2, and TYMS expression levels in young and aged ARDS mouse models,n = 6 in each group.The data are shown as mean ± standard deviation, and asterisks (*p<0.05, **p<0.01, ***p<0.001, ****p<0.0001) denote statistical significance; "ns" indicates no significance.

The murine model of ARDS was established by administering LPS to both aged (18 months) and young (3 months) mice. Despite being challenged with a lower dose of LPS (2.5 mg/kg) compared to young adult mice (5 mg/kg), aged mice exhibited more severe lung injury (**Figures 9A, B**). At 72 hours after LPS administration, aged mice exhibited lung edema, a condition not observed in young adult mice (**Figure 9C**). To confirm the mRNA expression levels of hub ARDS-ARDEGs, qRT-PCR was performed on total mRNA extracted from lung tissues (**Figures 9D–G**). After ARDS modeling, the expression of characteristic genes CKAP2, P2RY14, RBP2, and TYMS was significantly altered. Notably, there was a significant disparity in TYMS expression between the young and aged mice after modeling. The findings demonstrated that the four characteristic genes were strongly activated following the onset of ARDS, whereas aging inhibited the proper induction of TYMS expression in the progression of ARDS.

### 3.8 Difference analysis and GSEA analysis of TYMS grouping

**Figure 10:**
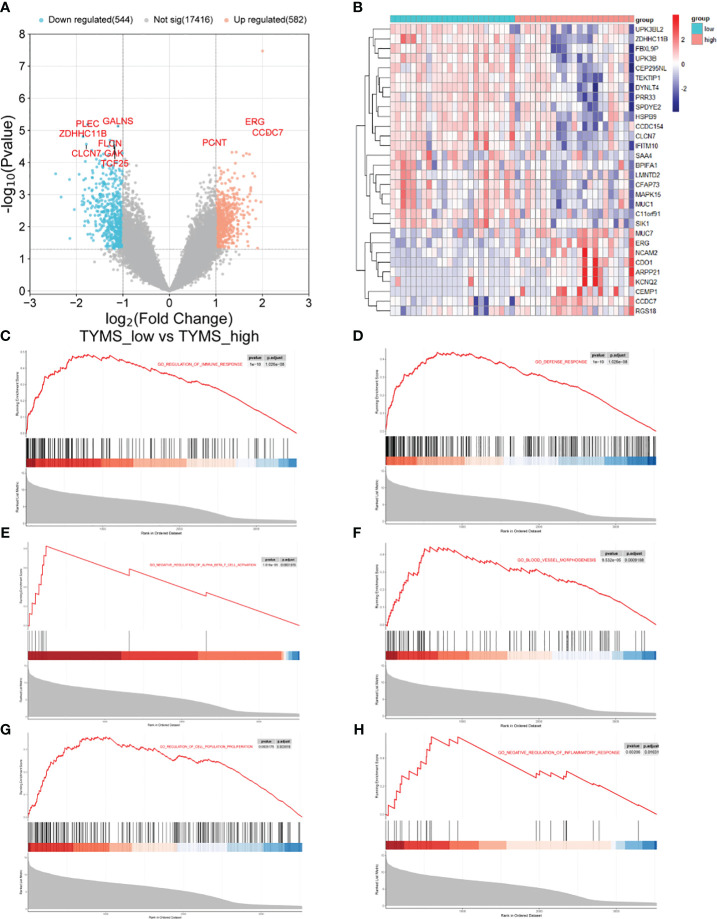
The GSEA of different TYMS expression groups in ARDS patients. **(A)** A volcano plot shows the differential gene analysis results between the ARDS patient groups with high and low TYMS expression. **(B)** A cluster heat map, ordered by *p*-value, displaying the top 30 genes linked to TYMS that are differentially expressed. **(C–H)** In the TYMS high expression group of ARDS patients, enrichment analysis reveals the upregulation of signaling pathways involved in the regulation of immune system processes, defense response, negative regulation of alpha-beta T cell activation, blood vessel morphogenesis, regulation of cell population proliferation, and negative regulation of inflammatory response in the TYMS high expression group of ARDS patients.

Based on the median TYMS value and a significant threshold of “*P*<0.05” and “|log2FC|>1,” tracheal aspirate samples from ARDS patients in the GSE163426 dataset were classified into two groups: a high-expression group and a low-expression group. As a result, 582 genes exhibited up-regulation, and 544 genes displayed down-regulation (**Figure 10A**). The heat map depicted the top 30 DEGs (**Figure 10B**). To evaluate the signaling pathways linked to TYMS, GSEA analysis was conducted (**Figures 10C–H**). The findings demonstrated a significant correlation between TYMS and the regulation of immune system processes, defense response, negative regulation of alpha-beta T cell activation, blood vessel morphogenesis, regulation of cell population proliferation, and negative regulation of inflammatory response.

### 3.9 Evaluation and analysis of immune cell infiltration

**Figure 11:**
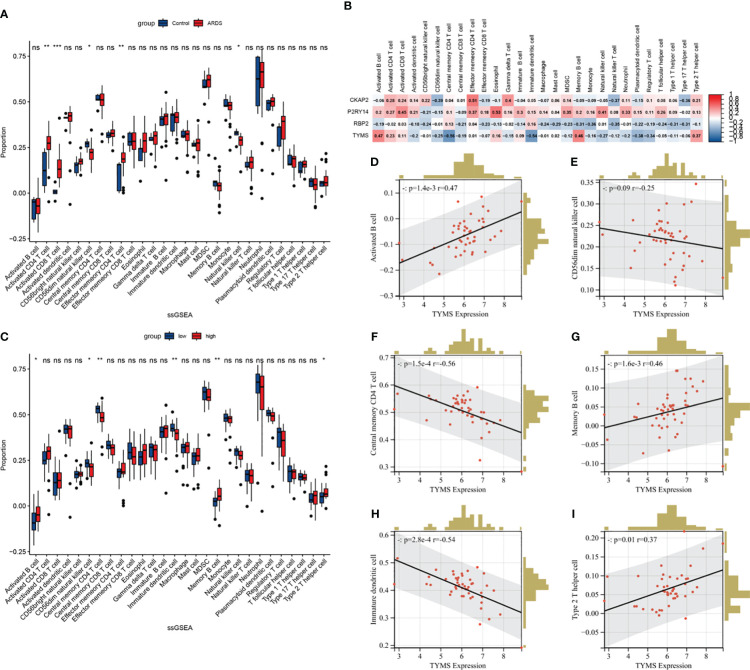
The ssGSEA immune infiltration analysis in ARDS patients. **(A)** Boxplot showing the difference in immune cell infiltration between controls and ARDS patients (controls vs. ARDS patients). **(B)** Heatmap depicting the correlation between hub ARDS-ARDEGs expression and different immune cell infiltrations. **(C)** Boxplot showing the differences in immune cell infiltration between high and low expression groups of ARDS patients according to TYMS expression. (high versus low). **(D–I)** The proportion of activated B cells, memory B cells, type 2 T helper cells, CD56^dim^ natural killer cells, central memory CD4 T cells, and immature dendritic cells in ARDS patients correlated with TYMS’s expression levels. The significance levels are: *P < 0.05, **P < 0.01, ***P < 0.001, ****P < 0.0001.

The single-sample gene set enrichment analysis (ssGSEA) algorithm was employed to analyze immune cell infiltration, aiming to discern immunological features (**Supplementary Table S10**). In ARDS samples, the infiltration levels of Activated CD4 T cells, Activated CD8 T cells, and Effector memory CD4 T cells were significantly increased compared to the healthy control. Conversely, the infiltration of CD56^dim^ natural killer cells and Natural killer cells in ARDS samples was significantly reduced (**Figure 11A**). Following this, a correlation analysis was conducted, linking the expression of hub genes with immune cell infiltration in ARDS patients (**Figure 11B**). Notably, a strong correlation exists between TYMS expression and the infiltration of various immune cells. Subsequent analysis involved grouping individuals based on TYMS expression levels to explore the relationship between TYMS and immune cell proportion (**Figure 11C**). The analysis revealed a significant upregulation in the proportion of activated B cells, Memory B cells, and Type 2 T helper cells in the group exhibiting high TYMS expression.

Conversely, there was a distinct decrease in the proportion of CD56^dim^ natural killer cells, Central memory CD4 T cells, and Immature dendritic cells. **Figures 11D–I** demonstrate the six correlations between TYMS and the immune cells. These findings indicate an association between TYMS and the regulation of immune responses, thereby enhancing the ability to resist pathogenic microorganisms and inhibiting excessive inflammatory responses.

### 3.10 Discrimination of cell source of TYMS at single-cell resolution

**Figure 12:**
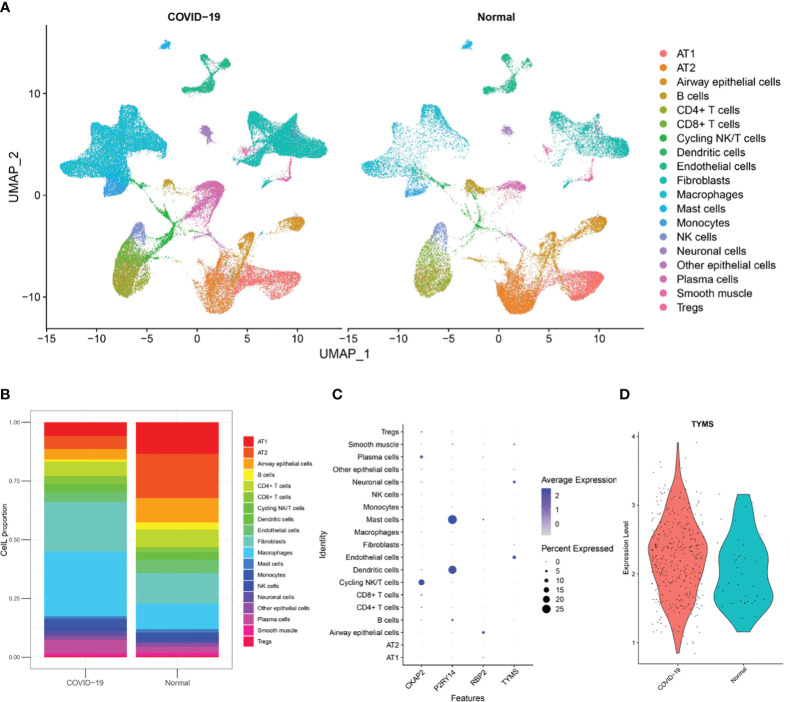
Analysis of single-nuclei RNA-Seq data. **(A)** A uniform manifold approximation and projection map showing the 19 cell types found using unsupervised analysis. The data is sourced from post-mortem lung tissue samples of COVID-19 fatal cases and seven healthy donors. **(B)** The 19 cell type proportions. **(C)** Dot plot illustrating the hub ARDS-ARDEGs expression levels in several cell types. **(D)** A violin plot showing how COVID-19 patients and healthy donors differ in the expression of TYMS in endothelial cells.

To investigate the cellular origin of TYMS, we acquired snRNA-Seq data, including data from autopsy lung tissues of approximately 116,000 nuclei taken from the lungs of nineteen individuals who died from COVID-19 and seven control individuals from GSE171524. Based on the clinical information of these 19 patients, it is evident that they were over 55 and presented with concomitant respiratory symptoms. Unsupervised analysis identified 19 distinct cell clusters (**Figure 12A**). These clusters were recognized as distinct cell types based on the expression levels of established markers: alveolar type I (AT1) cells, alveolar type II (AT2) cells, airway epithelial cells, B cells, CD4^+^ T cells, CD8^+^ T cells, cycling NK/T cells, dendritic cells, endothelial cells, fibroblasts, macrophages, mast cells, monocytes, NK cells, neuronal cells, other epithelial cells, plasma cells, smooth muscle cells, and Tregs. The disruption of alveolar epithelial and endothelial barriers was attributed to the loss of AT1 cells, AT2 cells, and endothelial cells and the proliferation of mononuclear/macrophage cells, fibroblast cells, and neuronal cells (**Figure 12B**). It was discovered that endothelial cells exhibited high TYMS expression (**Figure 12C**). Compared to the control group, patients with respiratory symptoms manifested a discernible upregulation in TYMS expression within endothelial cells (**Figure 12D**). The findings above indicate that TYMS is primarily expressed in endothelial cells, and moderate induction may contribute to endothelial regeneration and the maintenance of endothelial barrier integrity, thereby reducing the infiltration of inflammatory cells and inhibiting the progression of ARDS.

### 3.11 Investigation of communication between endothelial cells and other cell types

**Figure 13:**
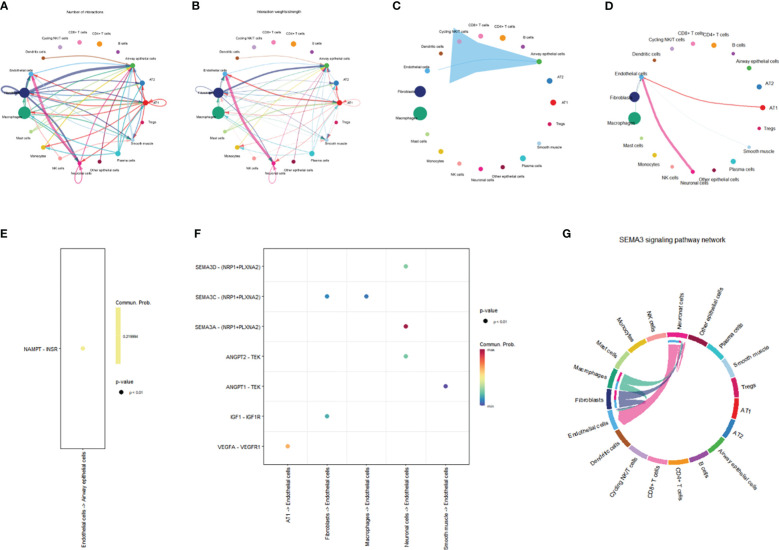
Network representation of cell-cell communication. **(A)** Frequency-based network diagram demonstrating the varying degrees of interactions among different cell types. **(B)** Intensity-based network diagram showcasing the strength of interactions among diverse cell populations. **(C)** Communication pathways and their associated intensities originating from endothelial cells are illustrated in a network diagram. **(D)** Communication pathways and their respective strengths through which endothelial cells receive signals are visualized in a network diagram. **(E)** Scatter plot detailing the ligand-receptor signaling patterns emitted by endothelial cells. **(F)** Scatter plot depicting the ligand-receptor signaling received by endothelial cells. **(G)** Chord diagram illustrating the intricate SEMA3 signaling pathway.

In order to evaluate intercellular communication, expression levels of ligands and their matching receptors are examined. The results above indicate that TYMS is primarily expressed in endothelial cells and is associated with immune cell infiltration. To investigate intercellular communication further, we employed the “CellChat” software package for a comprehensive analysis. Circular plots were generated to visualize the secretion signals involved in intercellular communication across all cell types (**Figures 13A, B**). The analysis demonstrated that endothelial cells communicate with airway epithelial cells through the VISFATIN signaling pathways (**Figure 13C**). Moreover, endothelial cells primarily receive signals from AT1 cells, fibroblasts, smooth muscle cells, neuronal cells, fibroblasts, and macrophages (**Figure 13D**).

Furthermore, we conducted a ligand-receptor pair analysis that identified specific signaling interactions. Endothelial cells were found to preferentially send signals via the NAMPT - INSR pathway while receiving signals through the VEGFA - VEGFR1, IGF1 - IGF1R, ANGPT1 - TEK, SEMA3A - (NRP1+PLXNA2), SEMA3C - (NRP1+PLXNA2), and SEMA3D - (NRP1+PLXNA2) pathways (**Figures 13E, F**). Intercellular communication between endothelial cells and other cells is predominantly mediated by the SEMA3 signaling pathway, with endothelial cells serving as the primary receiver cells in this pathway (**Figure 13G**). The SEMA3 signaling pathway exerts a significant influence on the vascular system, contributing to the development and regeneration of blood vessels. Conclusively, these discoveries provide invaluable perspectives on the intercellular dialogue between endothelial cells and various cell types, shedding light on the distinct signaling pathways and molecules implicated in this intricate process.

## 4 Discussion

ARDS is a common and severe respiratory condition, particularly in the elderly (35). Extensive research has established a close association between ARDS and the patient’s systemic or localized inflammatory response, with impairment of the pulmonary endothelial barrier function significantly contributing to lung injury and poor prognosis in sepsis and ARDS (36). Accumulating evidence also emphasizes the role of aging in this context, as ARDS incidence and mortality rates are increased in the elderly population (≥65 years old) (37). Research conducted by Soo Jung Cho et al. suggests that the susceptibility of the elderly to pulmonary diseases may be related to age-related changes in the composition and functionality of endothelial cells (11). Relevant studies have found elevated levels of ATP2B1 expression in ARDS patients, which is associated with endothelial barrier disruption (38). Aging impairs lung resident endothelial cell-mediated endothelial regeneration, leading to persistent inflammatory lung injury and high mortality rates (39). Aging exposes the immune system to sustained immune stressors and inflammatory assaults, contributing to immune senescence (40). These findings provide a solid basis to support the potential link between aging and immune regulation in ARDS.

With the current application of bioinformatics in the field of medicine, new avenues have opened up for scientific research on ARDS, allowing for the discovery of potential essential target genes. This study combines bioinformatics analysis with machine learning strategies to investigate the pathogenesis of ARDS from an aging perspective.

Through our analysis of normal and ARDS patient samples, we have identified 134 ARDEGs. Based on the results of GO and KEGG enrichment analyses, these ARDEGs are primarily involved in cellular responses to DNA damage stimuli, inflammatory reactions, negative regulation of innate immunity, and the FoxO signaling pathway and cellular senescence pathway. Our research highlights the involvement of these genes in inflammation response and aging. Increasing evidence supports the influence of aging on the occurrence of ARDS. For instance, elevated levels of reactive oxygen species production enzyme NADPH oxidase 4 in aging mice enhance endothelial cell permeability, impairing endothelial barrier function (41).

Additionally, KEGG pathway enrichment analysis indicates that these ARGs predominantly participate in the FoxO signaling pathway and lifespan regulation. FOXO transcription factors are crucial determinants of aging and longevity (42). Further research is needed to explore the hub ARDS-ARDEGs.

We identified four hub ARDS-ARDEGs (CKNP2, P2RY14, RBP2, and TYMS) using WGCNA analysis and three machine learning screenings (LASSO, RF, XGBoost). Our results indicated that four hub ARDS-ARDEGs were considerably elevated in samples from ARDS patients and had a robust diagnostic ability to predict ARDS.

CKAP2 (Cytoskeleton-associated protein 2, a crucial protein that controls cell proliferation), particularly during mitosis and cytokinesis (43). CKAP2 is up-regulated in a variety of malignant tumors and has diagnostic value in osteosarcoma (44), triple-negative breast cancer (45), gastric cancer, and other tumors (46). It has been reported that the CKAP2 gene plays a role in cell senescence (47), but its pathway needs to be confirmed by further studies. TYMS (Thymidylate synthase) is a gene that plays a crucial role in DNA replication and repair (48). Chen Y reported that the knockdown of TYMS resulted in reactive oxygen species generation, DNA damage, and cellular senescence (49). Restoring the expression and activity of FoxM1 may potentially enhance the functionality of endothelial cells and promote vascular neogenesis and repair (50). Additionally, as a downstream target of FoxM1, TYMS could be involved in the endothelial cell repair process (51). RBP2 (Retinoblastoma binding protein 2) facilitates the uptake, absorption, and metabolism of retinol (52). Moreover, it is implicated in the development of obesity and associated metabolic disorders (53). Several studies indicate that RBP2 plays a central role in maintaining innate immunity in the intestinal tract (54). Nevertheless, extensive research is still required to attain a comprehensive understanding of RBP2’s role in ARDS pathology. P2RY14, a G-protein coupled purinergic receptor, manifests its expression within the placenta, adipose tissue, intestine, stomach, and lung (55). Under conditions of tissue stress, alterations in the expression levels of P2Y14 occur, thereby influencing the processes of cellular aging and apoptosis (56). The expression of P2Y14R in human alveolar epithelial type 2 cells and its impact on IL-8 secretion and neutrophil recruitment suggest its pivotal role in the activation of airway epithelial cells and modulation of immune response (57).

To further validate the results of the bioinformatics analysis, we conducted qRT-PCR to identify and screen the four hub genes using lung tissue samples from both the control and ARDS model groups of mice. Remarkably, we observed significantly higher expression levels of all four genes in the lung tissues of ARDS mice, aligning with the findings of the bioinformatics analysis. Notably, there was a significant difference in TYMS expression between elderly and young mice after modeling, suggesting its potential as a diagnostic biomarker for elderly ARDS patients. Therefore, our subsequent research will focus on TYMS as the target gene for further exploration.

To investigate the possible biological functions and pathways TYMS may be involved in ARDS, we divided the ARDS dataset GSE163426 into groups based on the expression levels of TYMS and performed GSEA. The results revealed that TYMS plays a critical role in ARDS development by influencing immune inflammation and vascular morphogenesis. These findings are consistent with previous research on ARDS. We then conducted further research to evaluate the immunological infiltration in ARDS utilizing ssGSEA. In ARDS samples, we discovered a substantial increase in the infiltration levels of effector memory CD4 T cells, activated CD8 T cells and activated CD4 T cells. Additionally, TYMS demonstrated a negative correlation with CD56^dim^ natural killer cells, Central memory CD4 T cells, and immature dendritic cells, and a positive correlation with activated B cells, memory B cells, and Type 2 T helper cells by correlation analysis with immune cells. It has been established that inflammatory and immune-infiltrating cells, such as Type 2 T helper cells, which are mainly responsible for producing IL-4, IL-5, and IL-13 involved in humoral immunity, serve a purpose in the development of ARDS (58).

However, the intricate interaction among these immune cells, their distinct roles in ARDS, and the intricate molecular mechanisms involved still require further investigation for a comprehensive understanding.

Furthermore, we investigated the cellular localization of TYMS based on snRNA-Seq data. The results revealed different proportions of AT1 cells, AT2 cells, endothelial cells, mononuclear/macrophage cells, fibroblast cells, and neuronal cells between the control group and COVID-19-ARDS patients. Remarkably, the proportion of endothelial cells in ARDS patients was significantly lower compared to the healthy control group, indicating impaired endothelial cell function in ARDS patients. The increased proliferation of B cells is associated with improved survival rates in ARDS (59). Additionally, there was a decrease in alveolar epithelial cells in ARDS patients, consistent with the findings of a study by Feng et al. (60). TYMS showed high expression in the endothelial cells of ARDS patients, and moderate induction may contribute to endothelial regeneration and the maintenance of endothelial barrier integrity, thereby reducing the infiltration of inflammatory cells and inhibiting the progression of ARDS.

Within the microenvironment of the tissue, intercellular communication among diverse cell types assumes a consequential role. The results of this study demonstrate that endothelial cells communicate with airway epithelial cells through the VISFATIN signaling pathway. Additionally, endothelial cells receive ligand signals from other cell types, including AT1 cells, fibroblast cells, smooth muscle cells, neurons, fibroblast cells, and macrophages. The SEMA3 signaling pathway mainly mediates cell-to-cell communication between endothelial cells and other cells. The SEMA3 signaling pathway significantly impacts the vascular system, promoting vascular development and regeneration (61). In conclusion, these findings provide valuable insights into the intercellular communication between endothelial cells and other cell types, elucidating the specific signaling pathways and molecules involved.

Our research has limitations. Firstly, information from open databases served as the foundation for all analysis. The species representation, sequencing platforms, molecular types, sample grouping, and sample quality of the GEO collection are all limited. Nevertheless, the datasets utilized in this investigation were the only ones readily available for our analysis. Even though our cohorts have been constructed and confirmed, prospective research with additional validation would be ideal. Extensive research is also required to investigate the possible roles of hub genes in the pathophysiology of ARDS.

## Conclusion

Our study utilized bioinformatics analyses and machine learning methods to identify four potential aging-related genes associated with ARDS. The essential genes CKAP2, P2RY14, RBP2, and TYMS were validated using animal samples to determine their expression levels in an animal model through the protective role of TYMS in maintaining the integrity of the pulmonary microvascular endothelial barrier, promoting endothelial cell regeneration, and restoring its function. It could be a novel approach to the management and prevention of ARDS. These discoveries have immediate therapeutic implications, opening the door to more accurate diagnoses and individualized treatment plans. In the end, our study provides important insights for further investigation into ARDS and its treatment modalities while laying the groundwork for experimental and clinical research in the future.

## Data availability statement

The original contributions presented in the study are included in the article/supplementary material. Further inquiries can be directed to the corresponding author.

## Ethics statement

The animal experimental processes were approved by the Ethics Committee of Hangzhou Hibio Technology Co., Ltd. (HB2311003). The study was conducted in accordance with the local legislation and institutional requirements.

## Author contributions

GL: Funding acquisition, Writing – original draft, Writing – review & editing. PG: Data curation, Formal analysis, Visualization, Writing – original draft, Writing – review & editing. KY: Conceptualization, Data curation, Formal analysis, Writing – original draft. WZ: Validation, Writing – original draft. HP: Validation, Writing – original draft.
